# Chinese herbal medicine Tangshen Formula treatment for type 2 diabetic kidney disease in the early stage: study protocol for a randomized controlled trial

**DOI:** 10.1186/s13063-019-3821-6

**Published:** 2019-12-21

**Authors:** De Jin, Wen-Jing Huang, Xiang Meng, Fan Yang, Qi Bao, Mei-zhen Zhang, Ya-nan Yang, Qing Ni, Feng-Mei Lian, Xiao-Lin Tong

**Affiliations:** 1 Guang’anmen Hospital, Chinese Academy of Chinese Medical Sciences, Beijing, 100053 China; 2 Beijing University of Chinese Medicine, Beijing, 100029 China

**Keywords:** Diabetic kidney disease, Traditional Chinese medicine, Treatment, Randomized controlled trial

## Abstract

**Background:**

Diabetic kidney disease (DKD) is the main cause of end-stage kidney disease and has become a heavy economic and social burden due to its high prevalence and morbidity. The most effective strategy is that patients with DKD should be diagnosed and treated early. Preliminary studies showed that the Chinese herbal Tangshen Formula (TSF) may delay the progression of DKD, reducing microalbuminuria and macroalbuminuria and improving renal function. We designed a randomized, double-blind, placebo-controlled trial to evaluate the efficacy of TSF in patients with DKD.

**Methods/design:**

This trial is a 13-center, randomized, double-blind, placebo-controlled study. A total of 632 participants will be randomized in a 1:1 ratio to an experiment group (TSF plus losartan) and a control group (placebo plus losartan). The trial cycle will last 24 weeks. The primary outcome will be the change in the urine microalbumin–creatinine ratio from baseline to week 24. The secondary outcome will be the change in the rate of progression to the clinical proteinuria period after intervention, the rate of urine microalbumin negative conversion, the rate of normal urinary microalbumin, the doubling rate of the baseline creatinine value and the glomerular filtration rate between the two groups. Safety in medication will also be evaluated.

**Discussion:**

We hypothesize that patients with type 2 diabetes in the early stage of DKD will benefit from TSF. If successful, this study will provide evidence-based recommendations for clinicians.

**Trial registration:**

ClinicalTrials.gov, NCT03009864. Registered January 2017.

## Background

Diabetic kidney disease (DKD) is the most common diabetic microvascular complication and the primary cause of end-stage kidney disease (ESKD). Pooled data from 54 countries revealed that more than 80% of ESKD arises from diabetes, hypertension or a combination of both. The prevalence of ESKD was also up to 10 times higher in people with diabetes than in those without [1]. Several studies showed that 20% of people with diabetes from the UK [2] and 40% of individuals with diabetes in the USA will develop chronic kidney disease (CKD), whereas 19% show signs of DKD stage 3 or higher [3]. It is responsible for 40% of patients experiencing dialysis after diagnosis of diabetes mellitus in the developed world [4]. Moreover, there has also been a continuous increase in the incidence of ESKD due to the increasing incidence of T2DM, notably in China, with 11.9% from the high incidence of type 2 diabetes [5]. As impairment in renal function progresses, it is bound up with a high risk of mortality, cardiovascular events and hospitalizations [1] and is associated with a heavy economic burden, with estimated average annual healthcare costs ranging from $4573 to $10,322 [6]. Some risk factors of DKD have been identified, such as ageing, hypertension and hyperglycemia [7, 8]. Current strategies for DKD aim to delay deterioration of renal function through actively controlling glucose, blood pressure and blood lipid levels, and via restraint of the renin–angiotensin–aldosterone system (RAAS) [6–8], such as using angiotensin-converting enzyme inhibitors (ACEIs) and angiotensin II receptor blockers (ARBs).

However, these therapies are difficult to achieve in all patients with DKD and stable results in the long term as well as these treatments may not reverse DKD [9, 10], and even with treatment using currently effective therapies, ESKD will finally occur in a proportion of patients, requiring a dialysis regimen and kidney transplant. Therefore, the most effective strategy is that kidney disease in patients with diabetes should be diagnosed and treated early. Patients with type 2 diabetes with microalbuminuria are at high risk of progression to overt renal disease, indicating that those patients easily enter the stage of clinical diabetic nephropathy (stage 4 or 5). In addition, not only ESKD but also premature cardiovascular events are predicted by microalbuminuria in diabetes [11–13], whereas effective intervention therapies for microalbuminuria could improve clinical outcomes in DKD.

Although ACEIs or ARBs showed evidence for decreasing microalbuminuria in patients with type 2 diabetes and nephropathy [14, 15], some adverse events (cough, rhinitis, hyperkalemia, acute kidney injury and angioedema) related to ACEIs or ARBs might cause poor adherence [1–16]. Therefore, it is imperative to seek effective renoprotective therapies.

Chinese herbal medicine (CHM) has long been widely used in China [17]. Numerous studies have demonstrated the biological activity and therapeutic mechanism of CHM [18–21]. Recent studies show that certain Chinese herbs have renoprotective effects, improving the glomerular filtration rate (GFR) and decreasing proteinuria, especially in patients with microalbuminuria [22–26]. This study is designed to investigate whether Tangshan Formula (TSF) may represent a potential remedy for slowing disease progression in early type 2 diabetic nephropathy. If positive, this work may provide an evidence-based medicine remedy for slowing or preventing the clinical progression of DKD.

## Methods/design

### Study design

**Fig. 1:**
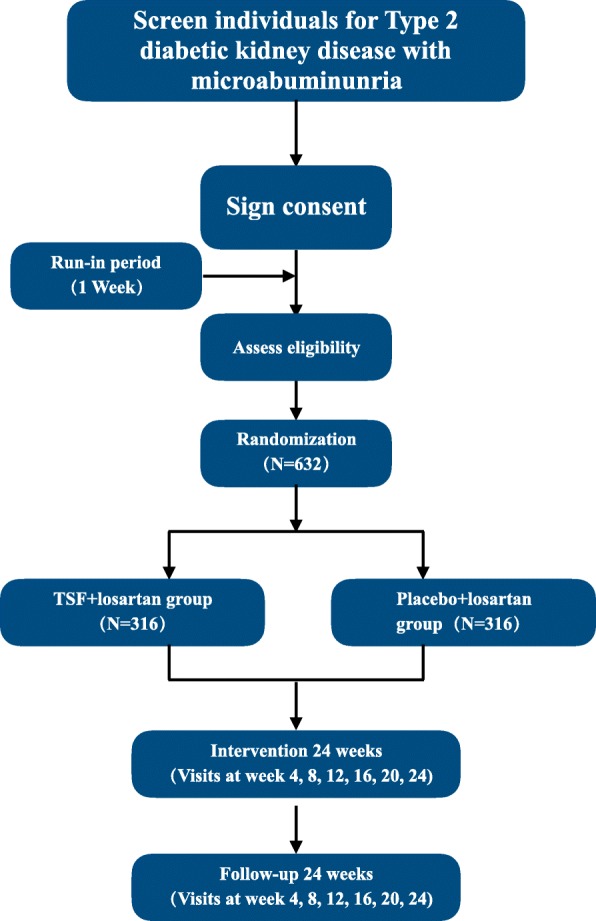
Flow diagram of enrollment, intervention and assessments. TSF Tangshen Formula

This protocol will be designed as a randomized, placebo-controlled and multicenter trial. Participants, investigators and statisticians will be blinded. A total of 632 subjects will be recruited at the following 13 tertiary A hospitals in mainland China: Guang An Men Hospital of the China Academy of Chinese Medical Sciences, The First Affiliated Hospital of Anhui University of Traditional Chinese Medicine, Beijing Hospital of Traditional Chinese Medicine, Hubei Hospital of Traditional Chinese Medicine, Zouping County Hospital of Traditional Chinese Medicine, Zibo Wanjie Hospital and Shijiazhuang Hospital of Traditional Chinese Medicine, Zhengzhou City Hospital of Traditional Medicine, Xingtai City Hospital of Traditional Medicine, Shexian Country Hospital of Traditional Medicine, Baoding City of Hospital of Traditional Medicine, Yantai Bai shi Hospital of Traditional Medicine and Jilin Province Hospital of Traditional Medicine. The trial will be implemented base on the principles of good clinical practice and reported according to the CONSORT statement [27, 28]. The trial flow diagram is illustrated in Fig. 1. The Standard Protocol Items: Recommendations for Interventional Trials (SPIRIT) [29] Checklist is shown in Additional file 1. This study has been registered at ClinicalTrials.gov (NCT03009864).

### Participants

#### Diagnostic criteria

The diagnostic criteria for this trial will be set based on the American Diabetes Association guidelines [7], Chinese Diabetes Society guidelines [8], National Kidney Foundation Kidney Disease Outcomes Quality Initiative (NKF-KDOQI) guidelines [30] and Kidney Disease Improving Global Outcomes (KDIGO) 2012 Clinical Practice Guideline [31]. For a diagnosis of early-stage DKD, patients with DM will have to present the following conditions:

1. Diagnosed with type 2 diabetes
2. Patients with type 2 diabetes with microalbuminuria and urinary microalbumin excretion rate (UAER)
  - For microalbuminuria, two repeated tests performed within 6 months will need to produce abnormal results
  - UAER of 30–300 mg/24 h or urinary albumin-to-creatinine ratio (ACR) of 30–300 mg/g (mg/mmol)
3. Have clinical manifestations of kidney diseases, such as edema, anemia, renal dysfunction, etc.

#### Inclusion criteria

1. Patient diagnosed with DKD with microalbuminuria
2. Age 30–70 years
3. Signed informed consent

#### Exclusion criteria

1. Proteinuria caused by non-DKD in the patient, such as gout, primary hypertension, tumors and proteinuria caused by chronic kidney disease
2. Cardiovascular, liver, kidney, hematopoietic system or other primary severe disease; serum transaminase more than double the standard value; serum creatinine (SCr) higher than the upper limit of normal; and psychiatric patients
3. Pregnancy, preparation for pregnancy or lactation, or any history of drug allergy
4. The patient developed renal failure (anemia and uremia)
5. Participation in another clinical trial or use of any other drug within the previous month
6. Recent use of ACEIs or ARBs except losartan in the past 1 month
7. Any excessive consumption of alcohol or any consumption of psychoactive substances, drug abuse or drug dependence during the past 5 years
8. According to the researcher’s judgment, as some other diseases or conditions reduce the possibility of enrollment or complicate the enrollment, such as frequent changes of jobs and an unstable living environment, which may easily lead to loss of visits

### Randomization and concealment

A specific randomization sequence will be computer generated by a computerized random number generator from an independent clinical research organization (CRO) of the Institute of Clinical Medicine of the Chinese Academy of Chinese Medical Sciences, which is not involved in the study. All eligible patients will be randomized to the experimental group or the control group at a 1:1 ratio. An independent non-investigator will protect the concealment list. The medical information will be confidential and not be available to any investigator for the duration of the study. If a medical emergency occurs, the individual’s randomization code and group allocation can be identified.

### Blinding

This study will be designed as double-blind. Not only are subjects and investigators blinded in these trials, but drug administration, statisticians and curative evaluators are also masked. Treatment allocation will be uncovered after completion of the study. In addition, TSF and placebo cannot be distinguished by their taste, smell and appearance. After production, study drugs will be packaged and transferred to numbered bottles by designated pharmacists in accordance with the randomized list. No one can tell the difference except the person who is in charge of concealment.

### Intervention

All subjects in the two groups will receive the conventional treatment, including oral losartan (50 mg/once a day), diet, exercise and oral medicine, to ensure access to steady levels of blood glucose, blood lipids and blood pressure, based on the recommendations of the American Diabetes Association guidelines [7] and Treatment of Chinese Diabetes Society guidelines [8]. Subjects will be randomly assigned to receive placebo (6 g/bag twice per day) or Tangshan Formula (6 g/bag twice per day) by specified sequence number from the central randomization system. The treatment will last for 24 consecutive weeks.

### Outcomes

#### Primary outcomes

Urinary microalbumin (MAlb) is a critical indicator for the diagnosis of early renal impairment in diabetes mellitus. For early-stage DKD, the urinary microalbumin–creatinine ratio (ACR) will be a primary evaluation index. The change in ACR from baseline will be evaluated between the two groups, and the ACR will be compared at baseline and treatment endpoint (24 weeks) in each group.

#### Secondary outcomes

1. Compare the ratio of progression into the clinical proteinuria period after intervention between the two groups
2. Urine microalbumin-negative conversion rate: the ratio of normal urinary microalbumin (< 20 μg/min) compared between the two groups
3. Change in GFR: change in the D-value and ratio of glomerular filtration rate (GFR) before and after treatment compared between the two groups. GFR calculated using the simplified MDRD formula: GFR (ml/min·1.73 m^2^) =186 × serum creatinine – 1.154 × age – 1.154 × [female × 0.742] × [Chinese × 1.233]
4. Doubling rate of baseline creatinine value compared between the two groups after intervention

##### Safety assessment

Adverse events (AEs) will be continuously monitored for 24 weeks at the beginning and end of the study period, and the incidence of AEs will be evaluated at each visit, including vital signs, ECG, liver function, renal function, routine blood examination, routine urinalysis and routine stool examination. In addition, AEs, such as signs and symptoms and other ailments, will also be documented truthfully at every study visit, including the occurrence time, severity, duration, effective measures and transfer. Each AE associated with the intervention drugs will be classified as mild, moderate and severe. Severe AEs will need to be submitted to the Principal Investigator and the ethics committee within 24 h. All AEs will be properly resolved. Criteria for the severity of adverse events’ detailed requirements are as follows:

- Mild: mild discomfort, subjects can endure no treatment, no special treatment is required, no effect on subjects’ recovery
- Moderate: moderate discomfort, unbearable to the subject, requiring special treatment, has a direct impact on the subject’s recovery
- Severity: severe discomfort, life-threatening, fatal or disabling, immediate emergency treatment is required

### Study visits and assessment

The intervention cycle will be 24 weeks, including a run-in period. After the research commences, visits will occur every 4 weeks during the study period. An overview of specific measurements and time points for data collection is presented in Table 1.

**Table 1:** Measurement items and point of data capture

Visit project	Screening period/baseline	Visits 1–2, 4–5	Visit 3	Visit 6
Visiting time	–7 to 0 days	Medication 4, 8, 16, 20 weeks ±7 days	Medication 12 weeks ±7 days	Medication 24 weeks ±7 days
Collect basic medical history				
Sign informed consent	×			
Fill in general information	×			
Medical history and treatment history	×			
Determine inclusion and exclusion criteria	×			
Vital signs	×	×	×	×
Physical examination	×	×	×	×
Comorbidity and medication records	×	×	×	×
Monitoring and inspection				
Urine pregnancy test	×			
Glycosylated hemoglobin, blood lipids	×		×	×
Fasting blood sugar and blood pressure	×	×	×	×
Effectiveness observation				
Urinary microalbumin–creatinine ratio	×	×	×	×
Urinary microalbumin excretion rate	×	×	×	×
Serum creatinine	×	×	×	×
Glomerular rate filtration	×	×	×	×
24-h urinary microalbumin and 24-h urinary microalbumin quantification	×		×	×
Physico-chemical examination				
Routine blood test and routine urine test	×		×	×
Stool routine examination	×		×	×
Vital signs	×	×	×	×
ECG, liver and kidney function	×		×	×
Adverse event		×	×	×
Other work				
Random grouping	×			
Distribute drug and patient journal cards	×	×	×	
Recovery drugs, quantity statistics		×	×	×
Recycle patient’s diary card		×	×	×
End of study summary				×

*ECG* electrocardiogram

### Quality control data collection

To maintain the high quality of this trial and ensure its adherence to the protocol, all investigators and drug administrators participating in the research will be trained rigorously based on a standardized operation practice (SOP) manual. Withdrawals or dropped visits will also need to be explained on case report forms (CRFs). All data will be documented on a standardized CRF and instantly recorded in the database via the ClinResearch Electronic Data Capture System (http://www.tcmcec.net/crivrs/). The monitor will review the CRFs, check the inclusion, exclusion and withdrawal criteria, as well as ensure information on the CRFs is in accordance with those in the source medical records. Original CRFs will be reserved at the research center for 5 years after completion of the study. The validity and authenticity of the multicenter trial will be guaranteed by establishing three committees, including the clinical trial guidance committee, the data and safety monitoring board and the outcome evaluation committee, each respectively being responsible for the trial design and executing the process, monitoring the data collection process to control its quality and evaluating the outcomes. The flow diagram of enrollment, interventions and assessments is shown in Fig. 1.

### Sample size

The sample size was estimated according to the relevant data from the losartan study published in *New England Journal of Medicine* [32, 33]. These results showed that the proportion of patients with the ACR reduced by 50% or more in the losartan group was 12.5% and the preliminary study of TSF data manifested that TSF as an add-on study can improve 50% of patients. The estimated proportion of patients with the ACR reduced by 50% or more was 18.7%. The estimated sample size formula was tested using the hypothesis of two population rates:

$$ n={\frac{\left[ u\alpha \sqrt{2p\left(1-p\right)}+ u\beta \sqrt{p1\left(1-p1\right)+p2\left(1-p2\right)}\right]}{{\left(p1-p2\right)}^2}}^2 $$

where *n* is the sample size; *p*_1_ and *p*_2_ are the sample rates, and *p* = (*p*_1_ + *p*_2_) / 2 is the sample average rate; and *α* is the type 1 error and *β* is the type 2 error, while *u*_*α*_ and *u*_*β*_ are the locus of the corresponding standard normal distribution. According to the unilateral test, *u*_*α*_ = 1.64485 and *u*_*β*_ = 0.84162, and substituting these into the formula presents *n* = 274.46. Therefore, 275 patients were needed in each group. Considering a dropout rate of no more than 15%, the final sample size was estimated to be 632 in total.

### Statistical analysis

The statistical analysis of this study was completed by an independent statistician, and the detailed statistical analysis plan was formulated separately by a statistician before the inception of this trial and determined with the Principal Investigator. Three analysis sets will be used for assessment of this study: the intention-to-treat set (ITT), the per-protocol analysis set (PPS) and the safety analysis set (SAS). The ITT and PPS will be used to appraise the efficacy of TSF. If any given case exists missing a critical variable, the last observation used as the final results will be carried forward to the final data. The changes of urinary microalbumin–creatinine ratio, urinary microalbumin, GFR, creatinine and baseline information will be present after treatment. A paired *t* test or the Wilcoxon signed-rank test will be employed to compare each group. Changes relative to baseline after treatment will be compared between groups using the *t* test or the Wilcoxon rank-sum test. *P* < 0.05 will be deemed to show a statistically significant difference.

### Bias analysis

The main evaluation outcome of this trial is the rate of progression from microalbuminuria into clinical proteinuria, which is very objective. Although ACEI and ARB drugs have achieved a recognized efficacy internationally, a certain proportion of patients still cannot delay the progression of DKD even using these two drugs. Therefore, the bias factors affecting the outcome evaluation include three aspects: blood glucose level; ACEI and/or ARB use; and laboratory testing error for urine microalbuminuria.

These factors are solved as follows: for the blood glucose level, the consistency of this factor in the two groups is ensured due to the random and double-blind study design method; subjects only take losartan and no other ARB or/and ACEI drugs; and central laboratories are used for the main outcomes.

## Discussion

DKD is part of the systemic microangiopathy and glomerular sclerosis caused by diabetes. In European and American countries, DKD is the primary cause of renal replacement therapy, accounting for about 1/2. It is the second common cause of ESRD in China after glomerular disease [1]. Compared to CKD from non-diabetic causes, DKD develops more rapidly into ESRD [34, 35]. It is urgent to seek an effective preventive measure to delay DKD. CHMs have been common in treating DM [36–41] and its complications [24, 42, 43], including DKD. In modern times, many patients have turned to Chinese herbal medicine for treatment as a complementary and necessary combination-drug therapy for kidney disease in China due to its fewer adverse reactions and more effective interventions. Currently, research from Taiwan has demonstrated that patients with CKD who used CHM had a significantly reduced ESRD risk (60%) [44]. Relevant clinical observations have shown that TSF appears to prevent further development and deterioration of the disease, which includes the reduction of urinary albumin and normalization of the glomerular filtration rate [45–47]. Potential mechanisms have proved that herbal medicine could regulate oxidative stress, which is well recognized to play a significant role in the worsening of DKD [45, 48, 49]. However, there was less clinical research on delaying the progression of DKD, especially in the early stage as the only period of reversing kidney lesions [50]. This work has the potential function to delay the development of DKD. Therefore, we are implementing this study to evaluate the efficacy and safety of TSF treatment for DKD. If successful, this work will provide evidence-based medical evidence for a therapeutic approach to delaying the progress of DKD.

### Trial status

Patient recruitment began in May 2018 and was expected to be completed in May 2022. At the time of manuscript submission, 95 patients had been recruited and the estimated time of recruitment completion is May 2020. Currently, we are still recruiting participants. Protocol version number 20,160,718 protocol02 (date 18 October 2016).

## Supplementary information

**Additional file 1.** SPIRIT 2013 Checklist: Recommended items to address in a clinical trial protocol and related documents.

## Abbreviations

ACEI: Angiotensin-converting enzyme inhibitor
ACR: Albumin-to-creatinine ratio
AE: Adverse event
ARB: Angiotensin II receptor blocker
CHM: Chinese herbal medicine
CKD: Chronic kidney disease
CRF: Case report form
CRO: Clinical research organization
DKD: Diabetic kidney disease
ESKD: End-stage kidney disease
GFR: Glomerular filtration rate
ITT: Intention-to-treat set
KDIGO: Kidney Disease Improving Global Outcomes
MAlb: Urinary microalbumin
NKF-KDOQI: National Kidney Foundation Kidney Disease Outcomes Quality Initiative
PPS: Per-protocol analysis set
RAAS: Renin–angiotensin–aldosterone system
SAS: Safety analysis set
SCr: Serum creatinine
SOP: Standardized operation practice
TSF: Tangshan Formula
UAER: Urinary microalbumin excretion rate
